# Postoperative Pain Profiles Associated With Pre-incisional Intravenous Paracetamol or Port-Site Infiltration of 0.5% Ropivacaine in Laparoscopic Cholecystectomy: An Observational Study

**DOI:** 10.7759/cureus.101975

**Published:** 2026-01-21

**Authors:** Afra Farheen Faiaz, Vidya Rani M, Mohammed Inayathulla Khan

**Affiliations:** 1 Anaesthetics, Chelsea and Westminster NHS Foundation Trust (WMUH), London, GBR; 2 Anesthesiology, Jagadguru Jayadeva Murugarajendra (JJM) Medical College, Davangere, IND; 3 Trauma and Orthopaedics, Barnet Hospital, London, GBR

**Keywords:** cholecystectomy, pain, paracetamol, ropivacaine, vas

## Abstract

Objective: This study aimed to describe and compare postoperative pain patterns associated with two commonly used pre-incisional analgesic practices: intravenous paracetamol and port-site infiltration of 0.5% ropivacaine in patients undergoing laparoscopic cholecystectomy.

Methodology: This observational study included 68 adult patients undergoing elective laparoscopic cholecystectomy under general anesthesia between December 2019 and April 2021. Ethical approval was obtained, and informed consent was secured from all participants. According to routine anesthetic practice, patients received either pre-incisional intravenous paracetamol (Group P, n = 34 (50%)) or port-site infiltration with 0.5% ropivacaine (Group R, n = 34 (50%)). Postoperative pain was assessed using the visual analog scale (VAS) at defined postoperative time points, and the need for rescue analgesia and the incidence of postoperative nausea and vomiting (PONV) were recorded.

Results: A total of 68 patients were analyzed, with comparable baseline characteristics between the intravenous paracetamol and port-site ropivacaine groups (mean age 42.5 ± 12.2 years; females 64.7%). Postoperative pain was observed in 48 (70.6%) patients, with a similar distribution between the two groups. Mean VAS scores declined from 7.55 ± 0.75 at 0 hour to 3.15 ± 1.4 at 24 hours in both groups. Rescue analgesia within 24 hours was required in 30 (44.1%) patients, more frequently in the intravenous paracetamol group (58.8%) than the port-site ropivacaine group (29.4%). Logistic regression showed higher, but non-significant, odds of rescue analgesia with intravenous paracetamol (OR 2.25; 95% CI 0.92-5.50).

Conclusion: Pre-incisional intravenous paracetamol combined with port-site infiltration of ropivacaine produces a predictable postoperative pain trajectory following laparoscopic cholecystectomy. Although the specific effect of port-site ropivacaine on local incision pain could not be isolated, the intervention contributes to overall pain control. No clear predictors of rescue analgesia were identified. These findings highlight the importance of incorporating site-specific analgesia into a broader multimodal pain management strategy to optimize postoperative comfort.

## Introduction

Laparoscopic cholecystectomy is widely performed because it is associated with reduced surgical trauma, earlier ambulation, shorter hospital stay, and lower postoperative morbidity compared with open procedures [1,2]. Despite these advantages, postoperative pain remains a relevant clinical concern, particularly during the early postoperative period, and effective pain management is essential to enhance recovery and patient comfort [3].

Postoperative pain following laparoscopic cholecystectomy is multifactorial, arising from several sources including port-site incisions, visceral manipulation, peritoneal irritation, and diaphragmatic stretching [4,5]. As a result, a variety of analgesic strategies are routinely employed in clinical practice, often as part of a multimodal analgesic approach. No single analgesic technique adequately addresses all pain generators associated with laparoscopic surgery, and anesthetic practice varies according to institutional protocols and clinician preference.

Pre-incisional administration of analgesic agents is commonly used in routine anesthetic practice to reduce postoperative pain intensity [4]. Although the concept of preemptive analgesia is well described, its clinical effectiveness depends on multiple factors, including the timing of administration, drug mechanism, and the type of surgical stimulus [6]. In real-world clinical settings, analgesic agents are frequently administered shortly before surgical incision as part of standard care, even when the ideal timing for preventing central sensitization cannot be ensured [7].

Paracetamol (acetaminophen) is a widely used analgesic and antipyretic agent with a favorable safety profile. Its analgesic effect is primarily mediated through central mechanisms, including inhibition of central prostaglandin synthesis and modulation of serotonergic pathways involved in pain perception [6]. Intravenous paracetamol is frequently incorporated into perioperative analgesic regimens because it allows reliable drug delivery when oral administration is not feasible.

Local anesthetic infiltration at surgical incision sites is another commonly employed technique for postoperative pain control. Ropivacaine, a long-acting amide local anesthetic, produces analgesia primarily at the site of administration, reducing nociceptive input from surgical wounds while minimizing motor blockade [7]. Port-site infiltration with ropivacaine is therefore frequently used to address incisional pain following laparoscopic procedures [8].

Both intravenous paracetamol and port-site local anesthetic infiltration are widely used in clinical practice; however, they act through different mechanisms and target various components of postoperative pain [9]. In many institutions, the choice between these techniques is influenced by clinician preference rather than direct comparative evidence derived from controlled trials [7].

The present observational study was undertaken to describe postoperative pain patterns and analgesic requirements associated with two commonly used pre-incisional analgesic practices: intravenous paracetamol and port-site infiltration of 0.5% ropivacaine in patients undergoing laparoscopic cholecystectomy. Given the differences in mechanism of action and the use of a global pain assessment tool, this study does not aim to establish comparative efficacy or causal superiority between the two interventions.

## Materials and methods

Study design and ethical approval

This single-center, observational, non-randomized study enrolled adult patients undergoing elective laparoscopic cholecystectomy under general anesthesia at a tertiary care hospital between December 2019 and April 2021. Ethical approval was obtained from Yenepoya Ethics Committee 2 (Approval No. YEC2/261), and written informed consent was obtained from all participants before inclusion.

Study population

Adult patients aged 18 to 60 years, of either gender, classified as American Society of Anesthesiologists physical status II or III [10], and scheduled for elective laparoscopic cholecystectomy were eligible. Patients were enrolled consecutively in accordance with routine institutional practice. Exclusion criteria included chronic pain, long-term analgesic or opioid use, known hypersensitivity to study medications, significant hepatic or renal dysfunction, and cases requiring conversion to open cholecystectomy.

Analgesic practices and group allocation

Group allocation was determined by the attending anesthesiologist as part of routine clinical care rather than randomization. Patients in the intravenous paracetamol group received 1 g of paracetamol administered after induction of anesthesia and before surgical incision. Patients in the port-site ropivacaine group received 20 mL of 0.5% ropivacaine evenly distributed across all port sites immediately before trocar insertion. The timing of administration followed standard operating room workflow; interventions are described as pre-incisional rather than mechanistically preemptive.

Anesthetic technique

All patients underwent standard intraoperative monitoring, including electrocardiography, non-invasive blood pressure measurement, pulse oximetry, and capnography. Premedication consisted of oral ranitidine 150 mg and lorazepam 1 mg administered the night before surgery. General anesthesia was induced using intravenous fentanyl at 1.5 μg/kg, along with other induction agents according to institutional protocol. Additional fentanyl doses were administered intraoperatively based on hemodynamic responses.

Anesthesia was maintained with isoflurane in a nitrous oxide-oxygen mixture (70:30). Nitrous oxide was used as part of routine institutional practice for laparoscopic surgery to reduce the required volatile anesthetic concentration and facilitate faster recovery. However, it is recognized that N2O and isoflurane may contribute to postoperative nausea and vomiting (PONV). End-tidal isoflurane concentrations were titrated to maintain adequate depth of anesthesia; exact minimum alveolar concentration (MAC) values varied according to patient response, reflecting real-world practice.

Postoperative pain assessment

Postoperative pain was assessed using a 10-cm Visual Analog Scale (VAS) representing global postoperative pain, including port-site discomfort [11]. Patients were familiarized with the VAS during pre-anesthetic evaluation. Pain assessments were conducted by trained nursing staff after patients had regained verbal responsiveness in the post-anesthesia care unit (PACU). VAS scores were recorded at PACU arrival (defined as 0 hours), 12 hours after PACU arrival, and 24 hours after PACU arrival. Pain assessment was global rather than site-specific; therefore, the direct effect of port-site ropivacaine on local incisional pain cannot be determined.

Rescue analgesia and postoperative nausea and vomiting

Rescue analgesia was administered when VAS exceeded 4 using intravenous morphine 2 mg, repeated as required according to institutional protocol. Time to first rescue analgesic administration within 24 hours was recorded. Episodes of PONV within 24 hours were documented as a descriptive outcome and were not considered a measure of analgesic efficacy.

Statistical analysis

Data were entered into a predesigned proforma and analyzed using SPSS version 23. Continuous variables are presented as mean ± standard deviation, and categorical variables as number and percentage (N (%)). Comparisons between groups were performed using Student’s t-test for continuous variables and chi-square test for categorical variables. A p-value < 0.05 was considered statistically significant. Logistic regression was performed to explore potential predictors of rescue analgesia, including group allocation, age, gender, BMI, ASA class, surgical time, previous abdominal surgery, and intraoperative opioid use. Analyses were intended to describe associations rather than establish causal relationships.

## Results

Baseline demographic and perioperative characteristics were comparable between the two groups. Females constituted 47 (69.1%) of the total study population, while males accounted for 21 (30.8%). Most patients were in American Society of Anesthesiologists class I, comprising 39 (57.4%) cases, followed by class II, with 29 (42.6%) cases. A history of previous abdominal surgery was present in 7 (10.3%) patients. Intraoperative opioid use was required in 11 (16.2%) patients. The mean age of the study population was 42.5 ± 12.2 years, with a mean body mass index of 25.5 ± 3.2 kg/m², and the mean surgical duration was 62.3 ± 13.5 minutes, showing comparable values between the intravenous paracetamol and port-site ropivacaine groups (Table 1).

**Table 1:** Baseline characteristics ASA: American Society of Anesthesiologists

Variable	IV Paracetamol (n=34)	Port-Site Ropivacaine (n=34)	Total (n=68)
Age (years), mean ± SD	39.7 ± 11.8	45.4 ± 12.1	42.5 ± 12.2
Female, n (%)	25 (73.5%)	22 (64.7%)	47 (69.1%)
Male, n (%)	9 (26.4%)	12 (35.3%)	21 (30.8%)
BMI (kg/m²), mean ± SD	25.3 ± 3.1	25.7 ± 3.2	25.5 ± 3.2
ASA class I, n (%)	20 (58.8%)	19 (55.9%)	39 (57.4%)
ASA class II, n (%)	14 (41.2%)	15 (44.1%)	29 (42.6%)
Previous abdominal surgery, n (%)	4 (11.8%)	3 (8.8%)	7 (10.3%)
Surgical time (min), mean ± SD	61.7 ± 12.9	62.9 ± 14.1	62.3 ± 13.5
Intraoperative opioid use, n (%)	6 (17.6%)	5 (14.7%)	11 (16.2%)

Pain was present in 48 (70.6%) patients overall, with 25 (73.5%) in the intravenous paracetamol group and 23 (67.6%) in the port-site ropivacaine group. Pain was absent in 20 (29.4%) patients, including 9 (26.5%) in the intravenous paracetamol group and 11 (32.4%) in the port-site ropivacaine group, with a comparable distribution of pain status between the two groups (Table 2).

**Table 2:** Pain presence at PACU arrival (including port-site discomfort) PACU: post-anesthesia care unit

Pain Status	IV Paracetamol (n=34)	Port-Site Ropivacaine (n=34)	Total (n=68)
Pain present	25 (73.5%)	23 (67.6%)	48 (70.6%)
No pain	9 (26.5%)	11 (32.4%)	20 (29.4%)

Global VAS scores demonstrated a progressive reduction in pain intensity over time in both groups. At 0-hour post-PACU arrival, the mean pain score was 7.6 ± 0.8 in the intravenous paracetamol group and 7.5 ± 0.7 in the port-site ropivacaine group, with an overall mean of 7.55 ± 0.75. At 12 hours, mean scores decreased to 5.9 ± 1.4 and 5.6 ± 1.4 in the respective groups, with an overall mean of 5.75 ± 1.4. By 24 hours, pain scores further declined to 3.2 ± 1.3 in the intravenous paracetamol group and 3.1 ± 1.5 in the port-site ropivacaine group, with an overall mean of 3.15 ± 1.4, indicating comparable pain relief trends over the postoperative period (Table 3).

**Table 3:** Global visual analogue scores (PACU 0, 12, 24 hours, including port-site pain component) Visual Analogue Scale (VAS) (© Physiopedia 2025) [11] PACU: post-anesthesia care unit

Time Post-PACU Arrival	IV Paracetamol, Mean ± SD	Port-Site Ropivacaine, Mean ± SD	Overall Mean ± SD
0 hour	7.6 ± 0.8	7.5 ± 0.7	7.55 ± 0.75
12 hours	5.9 ± 1.4	5.6 ± 1.4	5.75 ± 1.4
24 hours	3.2 ± 1.3	3.1 ± 1.5	3.15 ± 1.4

Rescue analgesia within the first 24 hours was required in 30 (44.1%) patients overall, with a higher proportion observed in the intravenous paracetamol group at 20 (58.8%) compared to the port-site ropivacaine group at 10 (29.4%). The mean time to first rescue analgesia was 298.2 ± 118.6 minutes in the intravenous paracetamol group and 319.4 ± 112.2 minutes in the port-site ropivacaine group, with an overall mean of 308.8 ± 115.6 minutes, indicating a longer time to first rescue analgesia in patients receiving port-site ropivacaine (Table 4).

**Table 4:** Rescue analgesia within 24 hours

Parameter	IV Paracetamol (n=34)	Port-Site Ropivacaine (n=34)	Total (n=68)
Patients requiring rescue analgesia, n (%)	20 (58.8%)	10 (29.4%)	30 (44.1%)
Time to first rescue analgesic (min), mean ± SD	298.2 ± 118.6	319.4 ± 112.2	308.8 ± 115.6
Postoperative nausea within 24 hours			
Yes	7 (20.6%)	12 (35.3%)	19 (27.9%)
No	27 (79.4%)	22 (64.7%)	49 (72.02%)

Logistic regression analysis was performed to identify predictors of rescue analgesia requirement within 24 hours. Patients in the intravenous paracetamol group had higher odds of requiring rescue analgesia compared with those receiving port-site ropivacaine (OR 2.25; 95% CI: 0.92-5.50), although this did not reach statistical significance (p = 0.073). Age, sex, body mass index, American Society of Anesthesiologists class, surgical duration, history of previous abdominal surgery, and intraoperative opioid use were not significantly associated with the need for rescue analgesia, with all predictors demonstrating non-significant p-values (Table 5).

**Table 5:** Logistic regression for rescue analgesia

Predictor	OR	95% CI	p-value
Group (paracetamol vs ropivacaine)	2.25	0.92–5.50	0.073
Age (per year)	0.98	0.94–1.02	0.321
Female vs Male	1.15	0.45–2.95	0.772
BMI (per kg/m²)	1.08	0.94–1.23	0.280
ASA II vs I	1.02	0.41–2.54	0.969
Surgical time (per min)	1.01	0.99–1.03	0.451
Previous abdominal surgery	1.35	0.32–5.62	0.680
Intraoperative opioid use	1.20	0.36–3.99	0.769

## Discussion

Preoperative planning for postoperative pain management should begin with patient education about the expected level of pain, the use of pain assessment tools, and the available analgesic methods [12,13]. Proper counseling helps alleviate patient anxiety and fears of inadequate pain control, thereby reducing the likelihood of postoperative discomfort. Patients should also be encouraged to communicate their analgesic needs clearly to ensure effective pain management [14].

Laparoscopic surgery aims to minimize surgical trauma while achieving satisfactory therapeutic outcomes. Effective postoperative pain control remains an essential component of anesthetic management in these procedures. Although local tissue infiltration has been practiced for decades, interest in this technique has recently re-emerged because of its favorable safety profile and consistent analgesic efficacy. Postoperative pain is multifactorial, arising from nociceptive, neuropathic, and inflammatory mechanisms [15].

Ropivacaine, a long-acting amide local anesthetic, was developed as a pure enantiomer. Its lower lipophilicity compared with bupivacaine reduces penetration of large myelinated motor fibers, resulting in reduced motor blockade. This characteristic makes ropivacaine particularly suitable for postoperative infiltration techniques, as it provides sensory analgesia with minimal motor impairment [16].

In the present study, the mean age of patients was 42.54 ± 12.21 years, with no significant difference between the two groups (39.7 ± 11.8 years in the paracetamol group vs. 45.4 ± 12.1 years in the ropivacaine group). These findings are consistent with those of Upadya et al., who also reported no significant age difference between the bupivacaine and paracetamol groups (41.93 vs. 38.46 years) [14].

A female predominance was observed, with 69.1% females and 30.8% males (female-to-male ratio: 2:1). This gender distribution parallels the trend reported in previous literature, including the findings of Upadya et al., who also observed a higher proportion of female patients (35 females vs. 25 males) [14].

Pain assessment was conducted using the VAS. Immediately postoperatively, pain was reported in 73.5% of patients in the paracetamol group, compared with fewer patients in the ropivacaine group. Similar patterns have been documented by Salihoglu et al., who found that paracetamol administration resulted in lower mean VAS scores than in untreated controls, suggesting that its analgesic effect, although modest, remains clinically relevant [16].

Although paracetamol is widely used for postoperative pain management, its delayed onset and the limited suitability of the oral route in the immediate postoperative period restrict its use as a sole agent. Intravenous administration overcomes these limitations. In our study, mean VAS scores at 12 and 24 hours did not differ significantly between the groups (p > 0.05), although the ropivacaine group consistently demonstrated slightly lower values. At 12 hours, mean VAS scores were 5.6 ± 1.4 in the ropivacaine group and 5.9 ± 1.4 in the paracetamol group; at 24 hours, the scores were 3.1 ± 1.5 and 3.2 ± 1.3, respectively. Although these differences are slight, the slightly lower VAS scores in the ropivacaine group may indicate a modest clinical benefit in patient comfort.

Gousheh et al. found that intravenous paracetamol (1 g) resulted in lower pain scores (VAS, p = 0.01) [17]. However, morphine consumption during the first six postoperative hours did not differ significantly between groups (p = 0.24) [17]. Their findings highlight that although paracetamol provides meaningful analgesia, it may not be adequate as a stand-alone agent for procedures associated with moderate pain.

The mean time to first rescue analgesia within 24 hours was longer in the ropivacaine group (319.4 ± 112.2 minutes) than in the paracetamol group (298.2 ± 118.2 minutes), but this difference was not statistically significant (p > 0.05). However, rescue analgesia was required significantly more often in the paracetamol group (58.8%) than in the ropivacaine group (29.4%) (p < 0.05), indicating better analgesic sustainability with port-site infiltration.

Similarly, Upadya et al. reported that patients receiving ropivacaine required delayed rescue analgesia and had lower mean VAS scores than those receiving paracetamol, supporting the superior analgesic efficacy and prolonged duration of action associated with ropivacaine infiltration [14].

Intravenous paracetamol 1 g has shown analgesic efficacy comparable to ketorolac 30 mg, diclofenac 75 mg, and morphine 10 mg in cases of moderate to severe postoperative pain. When combined with ketoprofen, it has reduced postoperative pain and total opioid use within 48 hours in double-blind, placebo-controlled trials [18-20]. Multiple clinical studies have demonstrated its opioid-sparing effect, with reductions in opioid consumption ranging from 24% to 46% and improved patient satisfaction [18-22]. A meta-analysis by Remy et al. further confirmed that paracetamol significantly reduces postoperative morphine requirements [23].

Limitations

This study has several limitations that should be considered when interpreting the findings. First, the sample size was relatively small (n=68), which may limit the generalizability of the results to broader populations. Second, the study was conducted at a single center, so institutional practices and variations in surgical technique may have influenced postoperative pain outcomes. Third, pain perception is inherently subjective and can be influenced by individual pain thresholds, psychological factors, and preoperative anxiety variables that were not assessed. Additionally, only short-term postoperative pain scores (up to 24 hours) were evaluated; extended follow-up may have provided a clearer picture of the duration of analgesic effectiveness. Finally, the absence of a placebo group or a combination-therapy group (intravenous paracetamol with local ropivacaine) limited the ability to assess potential additive or synergistic effects.

## Conclusions

Pre-incisional administration of intravenous paracetamol and port-site infiltration with ropivacaine were associated with a predictable postoperative pain trajectory following laparoscopic cholecystectomy. Pain assessment was global and included port-site discomfort, but the direct effect of port-site ropivacaine on local incision pain cannot be determined. No clear predictors of rescue analgesia were identified. These findings emphasize the importance of site-specific pain assessment in studies evaluating local anesthetic interventions and highlight that localized analgesia should be integrated into a broader multimodal pain management strategy.
